# A meal or a male: the ‘whispers’ of black widow males do not trigger a predatory response in females

**DOI:** 10.1186/1742-9994-11-4

**Published:** 2014-01-17

**Authors:** Samantha Vibert, Catherine Scott, Gerhard Gries

**Affiliations:** 1 Department of Biological Sciences, Simon Fraser University, 8888 University Drive, Burnaby, British Columbia V5A 1S6, Canada

**Keywords:** Sexual cannibalism, Sexual signalling, *Latrodectus hesperus*, Black widow spider, *Tegenaria agrestis*, Hobo spider, Vibration, Spider web

## Abstract

**Introduction:**

Female spiders are fine-tuned to detect and quickly respond to prey vibrations, presenting a challenge to courting males who must attract a female’s attention but not be mistaken for prey. This is likely particularly important at the onset of courtship when a male enters a female’s web. In web-dwelling spiders, little is known about how males solve this conundrum, or about their courtship signals. Here we used laser Doppler vibrometry to study the vibrations produced by males and prey (house flies and crickets) on tangle webs of the western black widow *Latrodectus hesperus* and on sheet webs of the hobo spider *Tegenaria agrestis*. We recorded the vibrations at the location typically occupied by a hunting female spider. We compared the vibrations produced by males and prey in terms of their waveform, dominant frequency, frequency bandwidth, amplitude and duration. We also played back recorded male and prey vibrations through the webs of female *L. hesperus* to determine the vibratory parameters that trigger a predatory response in females.

**Results:**

We found overlap in waveform between male and prey vibrations in both *L. hesperus* and *T. agrestis*. In both species, male vibrations were continuous, of long duration (on average 6.35 s for *T. agrestis* and 9.31 s for *L. hesperus)*, and lacked complex temporal patterning such as repeated motifs or syllables. Prey vibrations were shorter (1.38 - 2.59 s), sporadic and often percussive. Based on the parameters measured, courtship signals of male *L. hesperus* differed more markedly from prey cues than did those of *T. agrestis*. Courtship vibrations of *L. hesperus* males differed from prey vibrations in terms of dominant frequency, amplitude and duration. Vibrations of *T. agrestis* males differed from prey in terms of duration only. During a playback experiment, *L. hesperus* females did not respond aggressively to low-amplitude vibrations irrespective of whether the playback recording was from a prey or a male.

**Conclusions:**

Unlike courtship signals of other spider species, the courtship signals of *L. hesperus* and *T. agrestis* males do not have complex temporal patterning. The low-amplitude ‘whispers’ of *L. hesperus* males at the onset of courtship are less likely to trigger a predatory response in females than the high-amplitude vibrations of struggling prey.

## Introduction

Signals are shaped by the sensory system of the receiver and properties of the signal transmission channel. This “sensory drive” defines constraints imposed by each parameter on others [1,2]. In guppies, for instance, the light environment has shaped the visual system of the fish which, in turn, constrains the colour of males and their display behaviour toward females [3].

In web-dwelling spiders, the web is both a prey-capturing device and the signalling environment through which males transmit vibratory courtship signals [4-8]. This presents a challenge because most spiders are predatory and highly aggressive. Males are at risk of being treated as prey when they enter a female’s web and start signalling their presence. Indeed, sexual cannibalism in web-dwelling spiders has been widely documented [9-12]. This risk potentially constrains male courtship strategies.

Given that pre-copulatory cannibalism is never to a male’s advantage [11], a courting male must draw the female’s attention but minimize the risk of being attacked and consumed. In some species, males have evolved efficient strategies that help avoid cannibalism, such as cutting threads of the female’s web to limit her movement, courting from a mating thread [9,13], or attempting to mate with a moulting female [14].

The signal transmission properties of webs likely exert a strong influence on the male’s signalling strategy. Both the web and the female’s sensory system are fine-tuned to detect prey vibrations [15,16]. Males signalling with prey-like vibrations during courtship may be readily detected by females but may then face a predatory attack. Males avoiding prey-like vibrations may maximize their survival by clearly advertising themselves as potential mates. As yet little is known about how males signal their presence when they enter a female’s web [17,18], or about vibrations that entangled and struggling prey produce [15,19].

In our study we focus on the onset of courtship, when a male enters a female’s web. The identity-signalling challenge he faces is expected to occur in this early phase of courtship. Our study addresses web vibrations from the perspective of the female spider as the receiver of vibratory signals or cues, detecting vibrations from all areas of her web and from various sources. We document vibrations as they reach the female’s location after transmission through the web, rather than at the source, prior to transmission. We chose two species of web-dwelling spiders: the western black widow spider, *Latrodectus hesperus* Chamberlin and Ivie (Araneae: Theridiidae) which produces a tangle web, and the hobo spider, *Tegenaria agrestis* Walckenaer (Araneae: Agelenidae) which produces a sheet web. In both species courtship is lengthy (2–3 h for *L. hesperus* and 0.5-1.0 h for *T. agrestis*). The male’s courtship display takes place on the female’s web and comprises repeated behavioural elements that cause distinctive vibrations. In *L. hesperus*, females are much larger than males and exhibit aggression toward males in the early phase of courtship [20]. In *T. agrestis*, females are only slightly larger than males and are seldom aggressive towards them prior to copulation (S. Vibert, unpublished data).

Vibrations can be characterized by envelope (amplitude modulation), spectral (frequency) and temporal patterns (duration, periodicity of repeating elements). Information can be conveyed by all of these parameters [21]. To determine the parameter(s) triggering a behavioural response in a receiver, playback experiments have been widely used across taxa, including spiders [22,23], katydids [24], and tree frogs [25].

Our objectives were to (1) characterize vibrations produced by prey [house flies (*Musca domestica*); house crickets (*Acheta domesticus*)] and male spiders (*L. hesperus*, *T. agrestis*) during the first phase of courtship, at the female’s location; (2) determine whether male vibratory courtship signals differ from prey vibratory cues; and (3) determine the vibration parameter(s) that trigger a predatory response in females.

## Material and methods

### Characterization of prey and male vibrations on *L. hesperus* and *T. agrestis* webs

#### (a) Study animals

We chose our two study species because they are locally abundant and easily reared. We collected juvenile *L. hesperus* and *T. agrestis* from Island View Beach (48° 35' N, 123° 22' W, elevation 3–4 m), on the Saanich peninsula of Vancouver Island, British Columbia, Canada. We housed spiders individually in large Petri dishes (15 × 2.5 cm) at 20–25°C on a reversed 12:12 h (light:dark) light regime to facilitate experimentation during the spiders’ nocturnal activity phase. We raised spiders to adults on a diet of laboratory-reared house crickets and house flies*,* with water *ad libitum*. Like many spiders, *L. hesperus* and *T. agrestis* are generalist predators. They feed on various prey, both flying and crawling. We recorded vibrations produced by house crickets and flies in order to capture some of the diversity of prey vibrations to which *L. hesperus* and *T. agrestis* respond so that we could compare transmission characteristics of the same prey vibrations on two types of webs. Because adult *T. agrestis* females do not attack prey larger than themselves (S. Vibert, personal observation), we used nymphal 3-week-old crickets (mean mass: 27.1 mg (7.3 SD; *n* = 25)) and adult house flies (mean mass: 15.6 mg (4.3 SD; *n* = 25)) in our experiments. Both *T. agrestis* and *L. hesperus* females feed readily on these prey items and have been successfully reared on such a diet. Ten days post maturity, we placed virgin *L. hesperus* and *T. agrestis* females singly inside wood-framed boxes (30 × 30 × 20 cm and 15 × 20 × 15 cm, respectively) and allowed them to spin a web for 10 to 15 days. We tested a total of 27 and 18 webs in *L. hesperus* and *T. agrestis* trials, respectively, using virgin male spiders 7–10 days post maturity (mean mass (SD) of *L. hesperus* males: 22.6 mg (4.1); n = 21; of *T. agrestis* males: 154.0 mg (31.2); n = 17).

#### (b) Courtship behaviours

Both *L. hesperus* and *T. agrestis* males engage in the first (distal) phase of courtship in the absence of a female. Courtship of *L. hesperus* males consists of bouts of abdominal tremulations (dorso-ventral oscillations of the abdomen while stationary; see Additional file 1 for a video recording) and exploration of the web. Moreover, some males also cut some of the web’s threads, and bundle cut sections with their own silk [20]. Courting *T. agrestis* males engage in an extensive exploration of the female web. Their walking is always coupled with drumming with the pedipalps, tapping the web with the first pair of legs, and depositing silk (see Additional file 2 for a video recording). Occasionally, after several minutes of exploration, a male stands still and slowly drums with his pedipalps for a few seconds. Thereafter, he sometimes exhibits a “jerk”, or rapid contraction of all legs, and then immediately resumes walking while tapping and drumming (see Additional file 3 for a video recording). The jerks are a common part of the later, or proximal, phase of the courtship and are usually performed in close proximity to the female (S. Vibert, unpublished data), but are sometimes exhibited on an empty web. Additionally, a few of the males we observed stopped moving after bouts of walking while tapping and drumming and, while stationary, slowly contracted and relaxed all legs four or five times in succession (stretches).

#### (c) Recordings of web vibrations

We recorded web vibrations caused by prey or courting male spiders inside a sound-attenuated room on a concrete table to minimize extraneous acoustic or vibratory noise. Recordings employed a laser Doppler vibrometer (LDV; Polytec OFV-2500 with OFV-534 sensor head) and data acquisition software VIB-E-220 and VibSoft 4.8 (all products of Polytec Inc., Irvine, CA). Preliminary recordings with a 0 Hz to 2 kHz bandwidth did not reveal any prey or male vibrations with a dominant frequency ≥ 500 Hz. Thereafter, we acquired data with a 0 Hz to 1 kHz bandwidth and a frequency resolution of 78.125 mHz, applying no filtering. We limited all individual recordings to 12.8 s — the longest possible recording time under these settings. During tests with a male spider, we obtained simultaneous video recordings with a Canon FS100 camcorder (Canon USA, Lake Success, NY, USA).

**Figure 1:**
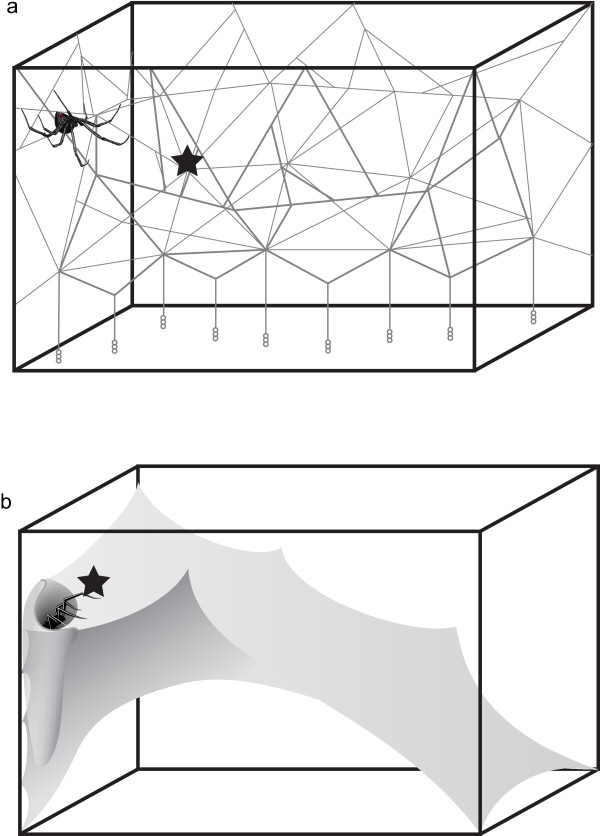
**Web structure of *Latrodectus hesperus *and *Tegenaria agrestis*.** Schematic drawing illustrating **(a)** the tangle web of *Latrodectus hesperus* and **(b)** the sheet web of *Tegenaria agrestis. L. hesperus* webs consist of a dense three-dimensional tangle of threads. Glue-coated capture threads extend from the tangle to the ground. *T. agrestis* webs consist of a two-dimensional sheet of silk with a funnel at one end serving as a retreat. We recorded vibrations on empty webs at the active hunting location of the spider (marked by ★). The spiders indicate the position of the female’s retreat. *L. hesperus* web illustration modified from Blackledge et al. 2005.

Prior to LDV recordings, we removed female spiders from their 10-15 day old webs. If we damaged a web in the process, we returned the spider to her web for 1–2 days to effect repairs. For recordings, we placed a small square (1 mm^2^; mean weight: 0.9 mg; n = 25) of reflective tape (Polytec Retroreflective Sheeting, Polytec, Inc.) on an empty web, at the top of the densest area of the tangle in front of the retreat (*L. hesperus*) and at the entrance to the funnel (*T. agrestis*) (Figure 1). These are the respective positions where spiders await prey. We then placed the box containing the web on a vibration-proof table, and focused the LDV beam on the reflective tape, at a 90° angle to the plane of the web in order to record transversal vibrations. Although we knew about the relevance of longitudinal and lateral web vibrations in other spider species [19], we restricted our measurements to transversal vibrations for technical reasons. The complex, 3-D structure of *L. hesperus* webs and the sheet-like nature of *T. agrestis* webs made it too difficult to position our equipment for recordings of lateral and longitudinal vibrations.

Before we introduced a prey or a male onto an empty web, we obtained a 12.8 s recording of background noise (the waveform and frequency spectrum of a representative background noise recording for each web type are presented in Additional file 4). Once we had dropped a 3-week-old cricket, or a house fly, onto a web, we commenced recordings of web vibrations as soon as the prey moved. We allowed prey to move freely within the enclosure containing the web. Most crickets quickly disentangled themselves from the web and then dropped to the bottom of the enclosure. Thereafter, many of the recordings captured vibrations produced by crickets coming into contact with capture threads. Thus, we recorded the vibrations that a waiting female would receive, after their transmission from all areas of the web. We recorded clips of 12.8 s in succession as long as the prey was moving. We terminated each test after 30 min or after 50 recordings. We report the mean number of vibration recordings obtained for each prey type in Additional file 5.

To record web vibrations caused by a courting male spider, we removed the male from his Petri dish and placed him in a 15 ml Falcon test tube for 2 h. Using forceps, we then gently placed the male on a randomly assigned web, starting concurrent LDV and video recordings as soon as he initiated courtship. We terminated each recording session after 30 min or when we had obtained 50 recordings. We limited our analyses to six randomly selected recordings per individual (see Additional file 5 for the number of recordings obtained for each individual prey or male).

#### (d) Analyses of LDV and video recordings

**Figure 2:**
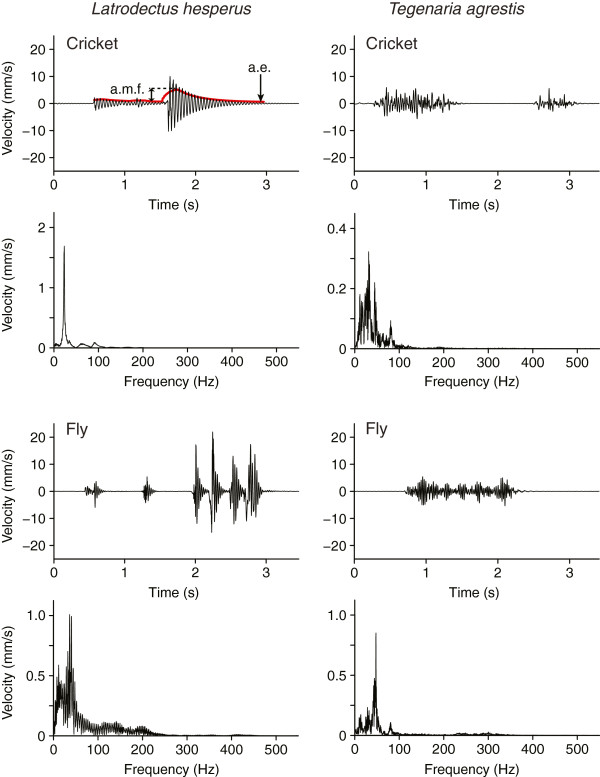
**Prey vibrations on webs of *Latrodectus hesperus *and *Tegenaria agrestis*.** Oscillograms depicting velocity [mm/s] over time [s] **(upper panels)** and frequency [Hz] **(lower panels)** of cricket and house fly vibrations recorded on empty webs of *Latrodectus hesperus* and *Tegenaria agrestis*. “a.e.” refers to root mean square amplitude envelope of the vibration and “a.m.f” refers to amplitude modulation factor measured between the lowest and the highest point of the amplitude envelope (in this example, a.m.f. = 120).

We exported the 12.8 s LDV recordings from VibSoft as WAV files, using the software Raven Lite 1.0 (Bioacoustics Research Program, Cornell Lab of Ornithology, Ithaca, NY) to determine recordings containing at least one ‘event’. We measured the maximum peak-to-baseline amplitude levels of background recordings and determined an amplitude threshold below which prey or male vibrations were indistinguishable from noise. We fixed this threshold at 75 μm/s. We define an ‘event’ as a prey or male vibration with an amplitude > 75 μm/s and lasting ≥ 0.2135 s. We ignored events ≤ 0.2135 s because reliable spectrograms could not be generated. We deemed an event to have ended when its amplitude dropped to below-threshold levels for at least 0.5 s. For each replicate of the cricket, fly, and male stimuli, we randomly selected six recordings with at least one ‘event’, and for each ‘event’ we measured six variables: (1) duration; (2) maximum peak-to-baseline amplitude, (3) root mean square (RMS) amplitude, (4) amplitude modulation factor (AMF), (5) dominant frequency, and (6) bandwidth. We note that vibrations of *L. hesperus* and *T. agrestis* males sometimes continued for more than 12.8 s (the maximum recording time), and that we could not include these lengthy vibrations in quantitative analyses. Nonetheless, our recordings were sufficiently long to detect a significant difference between the males’ vibrations and the much briefer prey vibrations (see Results). We calculated dominant frequency using a fast Fourier transform (FFT). We measured bandwidth as the range of frequencies with an amplitude above a threshold of ¼ the amplitude of the dominant frequency. For each replicate and for each quantitative variable, we calculated a mean value based on all events contained in the six recordings analysed. We report the mean number of events measured within six randomly selected recordings for each fly, cricket and male replicate in Additional file 5. We measured the RMS amplitude envelope for each vibration, using 0.2 s intervals [26]. As a measure of the “percussiveness” of recorded vibrations, we then calculated an amplitude modulation factor (AMF) by dividing the maximum envelope amplitude value by the minimum value. Low AMFs correspond to vibrations with small changes in amplitude, whereas high AMFs correspond to vibrations with large changes in amplitude (Figure 2).

We reviewed video recordings of the male’s courtship behaviour, which we acquired concurrently with LDV recordings, with Windows Movie Maker 6.0 (Microsoft Corporation). Matching the time frames between LDV and video recordings allowed us to link specific vibrations with the specific male behaviours listed above.

#### (e) Statistical analysis

To achieve normality and equality of variance of data, we subjected data to a Box-Cox transformation prior to analyses. For each species, we conducted a linear discriminant analysis to test whether recorded vibrations could be reliably assigned to their source (fly, cricket, or male) based on the vibratory parameters measured (dominant frequency, frequency bandwidth, RMS amplitude, and duration). We then conducted univariate ANOVAs for individual response variables, followed by Tukey’s HSD post-hoc analyses. We used JMP 8.0 (SAS Institute Inc.) for all statistical analyses.

### Vibration parameters *L. hesperus* females use to discern a prospective prey from a courting male

#### (a) Study spiders

We reared *L. hesperus* as described above. We kept virgin females ≥10 days post maturity singly inside wood-framed boxes (30 × 30 × 20 cm) and allowed them to spin a web for 21 days during which time they received four house flies per week. We did not feed spiders for seven days prior to testing to increase the probability that females would respond to a prey stimulus.

#### (b) Test stimuli

**Figure 3:**
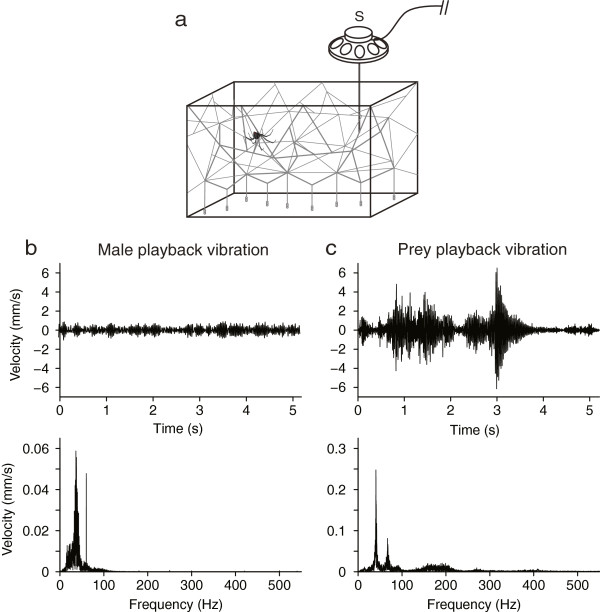
**Playback design and original vibrations used during playback. (a)** Schematic drawing of the experimental design for vibration playbacks. A looped male vibration and a looped house fly vibration were played back at low and high amplitude by a modified loudspeaker (S) placed in contact with the web 15 cm away from a *Latrodectus hesperus* female in her hunting position; **(b)** Original male abdominal tremulation vibration used to generate the input vibrations played back with the speaker. Oscillograms depict velocity [mm/s] over time [s] (upper panel) and frequency [Hz] (lower panel); **(c)** Original fly vibration used to generate the input vibrations played back with the speaker. Oscillograms depict velocity [mm/s] over time [s] (upper panel) and frequency [Hz] (lower panel).

From the vibrations obtained in the previous section we selected a prey and a male vibration for testing the females’ behavioural responses. The percussive type prey vibration (AMF = 34) had been generated by a house fly. The male vibration corresponded to a male’s abdominal tremulation (see Additional file 1 for a video recording of a male’s tremulation), with a rather constant waveform (AMF = 3). We use the term “waveform” to describe or refer to amplitude changes of a vibration over time. The prey vibration had a dominant frequency of 40 Hz with a secondary peak of 67 Hz. The male vibration had a dominant frequency of 36 Hz with a secondary peak of 60 Hz (Figure 3b,c). These two dominant frequency values are intermediate between the mean dominant frequency of vibrations produced by house flies (28.57 Hz), crickets (31.82 Hz), and by *L. hesperus* males (52.34 Hz). For bioassays, we extracted a 5.2 s segment of the selected prey or male vibration with Raven Lite 1.0 from original 12.8 s LDV recordings, and then looped it for 5 min of continuous playback in order to give females ample time to respond. As most females responded within 10 s (see Results), before two repetitions of the 5.2 s segment were complete, it is not likely that the looping of test stimuli altered the females’ responses.

Based on data obtained in the previous section, the mean maximum amplitude of male abdominal tremulations was 1.85 mm/s (2.35 SD; *n* = 17), whereas the mean maximum amplitude of fly vibrations was 21.25 mm/s (15.71 SD; *n* = 16). We modified amplitude levels of test stimuli with the “Amplify” function in Raven Lite 1.0 to produce a ‘low’-amplitude test stimulus equivalent to the mean amplitude of male abdominal tremulations (‘low’ mean maximum amplitude = 1.99 mm/s), and to produce a ‘high’-amplitude test stimulus equivalent to half the mean amplitude of fly vibrations (‘high’ mean maximum amplitude = 9.47 mm/s). Equipment limitations did not allow playback of vibrations at a higher amplitude. We measured the stimulus amplitude levels during a calibration experiment described in Additional file 6. We report details pertaining to the quality and consistency of playback test stimuli in terms of waveform and amplitude in Additional files 6 and 7.

#### (c) Playback

The playback apparatus (see Figure 3a) consisted of a modified unenclosed loudspeaker (12 Ω; 14 cm diam.) with its cone removed, and a metal rod (180 × 1 mm) attached to the centre of the dust cap. We attached the loudspeaker to an adjustable stand so that it was perpendicular to the plane of the upper tangle of the web, and the tip of the rod could make contact with several silken strands. We connected the speaker to an amplifier (Creek OBH-21SE) which we plugged into the headphone jack of a laptop computer (Toshiba Satellite, Pentium 4, 2.66GHz processor; operating Windows XP version 2002). On the laptop, we opened the looped ‘prey’ and ‘male’ vibration playback files with Windows Media Player 11.0, and played them through the speaker, resulting in vertical movements of the rod.

#### (d) Behavioural response of spiders to playback vibrations

Prior to testing, we inspected each web for the position of the spider. If she was not in her active hunting position (see above), we postponed testing until the following dark phase. If she was in a hunting position, we chose a location on the upper part of the tangle, 15 cm from her, for the input of playback vibrations. During the initial distal phase of courtship, males spend most of their time on the top part of the web and court far away from the female, usually at a distance between 10 and 30 cm. We selected a location on the web where a male would be likely to court and that was both accessible to the playback apparatus and connected to the female by a dense tangle of threads. We positioned the loudspeaker above the chosen location and put the rod in contact with the web, imposing minimal tension on the threads contacted. During playbacks, contact between the threads and the rod was maintained by this slight tension and the adherence of the silk. If the spider moved during positioning of the rod, we postponed testing for at least 1 h. Once the rod was in place, we started simultaneously the vibration playback (Windows Media Player 11.0) and behaviour-scoring software (JWatcher 1.0, [49]).

Upon entering a female’s web, a courting *L. hesperus* male invariably engages in lengthy and repeated bouts of abdominal tremulations. During the early (distal) phase of courtship, females are typically immobile and do not display any response to a courting male. When approached by a male, they sometimes respond by twitching their abdomen [20,27]. In contrast, females respond to the presence of a struggling prey on their web by rapidly moving toward the prey (S. Vibert, personal observation). We recorded the time of the spider’s first predatory response, which we defined as a forward motion of more than 1 cm toward the source of the vibration (see Additional file 8 for a video recording of a female’s response). Spiders that did not move at all, readjusted the position of their legs, oriented toward the rod without forward motion, or twitched their abdomen, were all scored as non-responders. We stopped the playback after 5 min or once the spider had reached the rod, whichever came first. We tested each of 64 spiders only once so that each of four treatments entailing ‘low’- or ‘high’-amplitude vibrations of prey or males (prey/low, prey/high, male/low, male/high) was replicated 16 times.

#### (e) Statistical analyses

We used a nominal logistic regression to test the effect of amplitude or waveform of playback vibrations, or interaction between these parameters, on the females’ predatory responses. We then used the latency of the females’ responses to conduct a survival analysis [28], with non-responders right-censored at 5 min, to determine whether response times of females within the ‘low’-amplitude level and the ‘high’-amplitude level differed based on waveform. We used JMP 8.0 for all analyses.

## Results

### Characterization of prey and male vibrations on *L. hesperus* and *T. agrestis* webs

**Figure 4:**
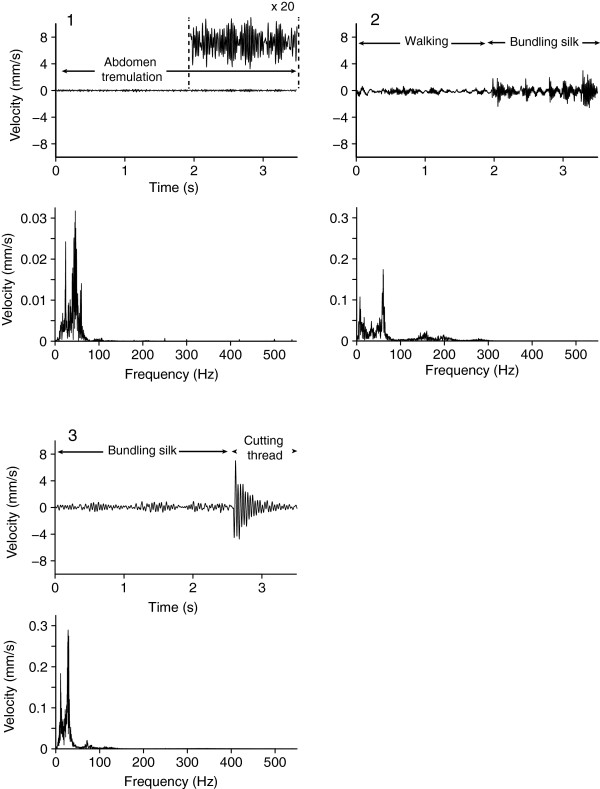
**Courtship vibrations of male *Latrodectus hesperus*.** For each of the courtship vibrations including **(1)** abdominal tremulations, **(2)** walking and bundling silk, and **(3)** bundling silk and cutting displayed by *Latrodectus hesperus* males on empty webs of conspecific females, the upper panel depicts vibrations in the time domain, and the lower panel depicts vibrations in the frequency domain. The insert in **(1)** depicts the amplitude of abdominal tremulation (maximum baseline-to-peak amplitude = 0.7 mm/s) magnified 20 times.

Parameters of prey and male vibrations are compiled in Table 1. On *L. hesperus* webs (Figure 1), cricket and fly vibrations were typically brief and percussive, with rapid and strong changes in amplitude (= high amplitude modulation factor (AMF); Figure 2). RMS amplitudes of fly vibrations were on average 3 times greater than those of cricket vibrations. Courting *L. hesperus* males, in contrast, typically produced continuous instead of intermittent vibrations that often persisted throughout the 12.8 s recording period (Figure 4). When cutting web threads, males produced brief, high-amplitude vibrations that resembled those of prey, but before and after cutting threads they typically produced continuous vibrations by walking or bundling silk. Their walking on webs and bundling silk produced sustained vibrations with varying amplitude (moderate AMF) but with no complex temporal pattern. Only four males engaged in cutting threads and bundling silk. Abdominal tremulation produced unique signals of very low and fairly constant amplitude (low AMF), and continuous duration (Figure 4).

**Table 1:** **Summary of parameters associated with males, prey, and background noise vibrations on webs of *Latrodectus hesperus *** **(top) and *Tegenaria agrestis *** **(bottom)**

	**Male**					**Prey**		**Background**
	**Abdomen tremulation**	**Walking**	**Bundling silk**	**Cutting**	**All**	**Cricket**	**Fly**	
Dominant frequency (Hz)	**43.38** (26.78)	**55.38** (31.65)	**36.58** (16.9)	**36.6** (16.77)	**52.34** (25.28)	**31.82** (16.29)	**28.57** (17.41)	**32.63** (16.41)
Bandwidth (Hz)	**43.38** (29.42)	**44.73** (39.85)	**44.96** (71.56)	**4.1** (2.79)	**74.8** (75.5)	**45.4** (34.57)	**54.73** (53.02)	
RMS amplitude (mm/s)	**0.46** (0.61)	**1.53** (0.94)	**1.32** (0.87)	**3.73** (3.09)	**0.60** (0.34)	**0.88** (0.83)	**3.27** (2.59)	**0.03** (0.01)
Max amplitude (mm/s)	**1.85** (2.35)	**14.44** (11.73)	**6.04** (4.05)	**17.91** (10.66)	**6.27** (4.42)	**7.58** (8.71)	**21.25** (15.72)	**0.10** (0.04)
A.M.F	**3.91** (1.20)	**14.69** (12.08)	**8.12** (5.55)	**11.50** (6.75)	**9.65** (9.55)	**33.78** (33.16)	**51.56** (48.07)	
Duration (s)	**6.45** (3.0)	**8.06** (4.12)	**8.59** (4.31)	**1.0** (0.04)	**9.31** (2.43)	**1.38** (1.0)	**2.01** (0.8)	
*n*	17	18	4	4	16	16	16	48
	**Palp drumming**	**Walking, drumming tapping**	**Jerk**	**Stretch**	**All**	**Cricket**	**Fly**	
Dominant frequency (Hz)	**32.83** (19.91)	**40.64** (18.56)	**50.88** (8.85)	**54.46** (25.79)	**44.31** (20.08)	**45.88** (28.11)	**40.57** (39.01)	**34.14** (20.58)
Band-width (Hz)	**92.63** (63.35)	**71.38** (18.75)	**63.35** (18.96)	**107.31** (113.6)	**65.34** (21.54)	**105.18** (85.82)	**54.39** (38.77)	
RMS amplitude (mm/s)	**0.21** (0.16)	**2.19** (1.98)	**3.52** (4.63)	**4.05** (3.88)	**0.86** (0.58)	**0.42** (0.30)	**1.81** (2.22)	**0.02** (0.01)
Max amplitude (mm/s)	**1.36** (1.04)	**20.37** (19.41)	**19.13** (25.91)	**28.54** (30.11)	**8.27** (5.86)	**2.92** (2.21)	**7.06** (6.0)	**0.05** (0.02)
A.M.F	**5.37** (2.91)	**29.95** (17.88)	**10.34** (3.72)	**7.49** (5.46)	**14.49** (14.85)	**23.04** (34.13)	**34.29** (42.0)	
Duration (s)	**7.25** (3.39)	**9.62** (3.17)	**1.58** (1.20)	**1.43** (1.24)	**6.35** (2.73)	**1.44** (0.47)	**2.59** (1.12)	
*n*	10	13	6	9	16	16	16	38

Means are in bold face and standard deviations are in parentheses.

**Figure 5:**
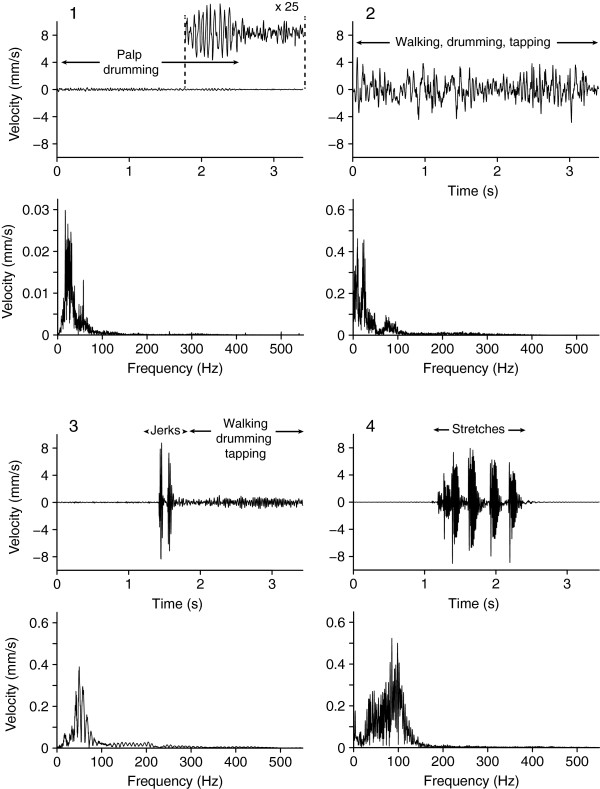
**Courtship vibrations of male *Tegenaria agrestis*.** For each of the courtship vibrations including **(1)** palp drumming, **(2)** walking, drumming and tapping, **(3)** jerks, walking, drumming and tapping, and **(4)** stretches produced by courting *Tegenaria agrestis* males on empty webs of conspecific females, the upper panel depicts vibrations in the time domain and the lower panel depicts vibration in the frequency domain. The insert in **(1)** depicts the amplitude of palp drumming (maximum baseline-to-peak amplitude = 0.3 mm/s) magnified 25 times.

On *T. agrestis* webs (Figure 1), cricket and fly vibrations resembled those on *L. hesperus* webs (Figure 2), with fly vibrations on average of greater amplitude than cricket vibrations. Some fly vibrations also contained a high-frequency component (~ 200 Hz) corresponding to wing beats. Courting *T. agrestis* males produced four distinct types of vibrations: (1) drumming with their pedipalps produced continuous, low-amplitude and low-amplitude-modulation vibrations unique to males; (2) walking on webs while pedipalp-drumming and tapping with the first pair of legs produced sustained vibrations of varying amplitude (high AMF); (3) jerks (see methods) produced brief and highly percussive types of vibrations that resembled those of prey but were always followed by continuous vibrations associated with walking on the web while drumming and tapping; and (4) stretches (see methods) produced a distinct temporal pattern of four or five percussive vibrations which were always preceded and followed by silence (Figure 5).

### Comparison of prey and male vibrations on *L. hesperus* and *T. agrestis* webs

**Figure 6:**
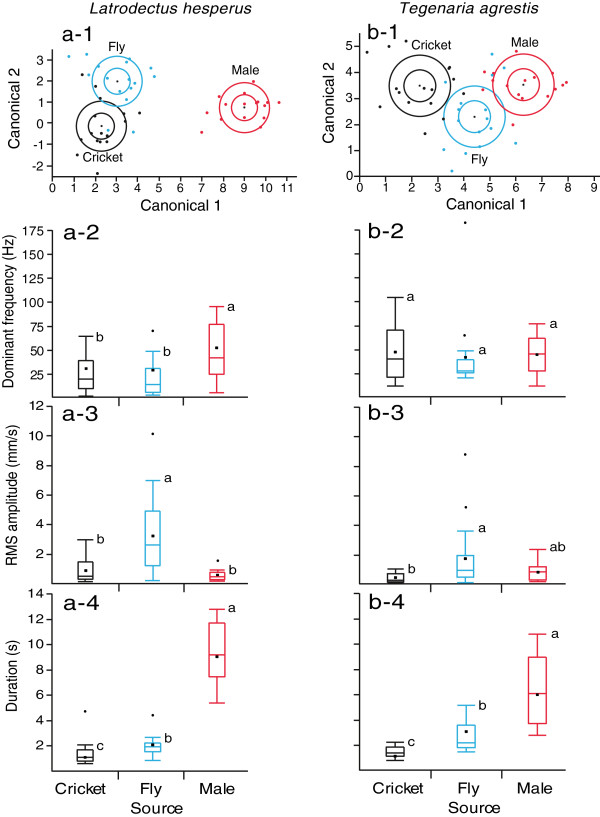
**Comparison of vibration parameters associated with struggling prey and courting male spiders on webs of *Latrodectus hesperus *and *Tegenaria agrestis*. (a-1)** and **(b-1)** Discriminant function analysis using dominant frequency, frequency bandwidth, root mean square amplitude and duration (transformed data) of vibrations produced by cricket and house fly prey and by males of *Latrodectus hesperus* and *Tegenaria agrestis* on empty *L. hesperus* webs **(a-1)** and *T. agrestis* webs **(b-1)** (n = 16 each). The inner circle shows the 95% confidence ellipse of each mean; the outer circle shows the normal 50% contours; **(a-2 to a-4)** and **(b-2 to b-4)** Boxplots of dominant frequency [Hz], root mean square (RMS) amplitude [mm/s] and duration [s] of vibrations produced by cricket and house fly prey and by males of *L. hesperus* and *T. agrestis* on empty *L. hesperus* webs **(a-2 to a-4)** and *T. agrestis* webs **(b-2 to b-4)** (n = 16 each). Median, mean, interquartile range (IQR) and outliers (untransformed data); whiskers = upper and lower data point values within 1.5 IQR; means with different letters are significantly different (Tukey’s HSD on transformed data, *p* < 0.05).

For webs of both *L. hesperus* and *T. agrestis* females, linear discriminant analyses revealed significant variation in dominant frequency, bandwidth, RMS amplitude, and duration of vibrations produced by prey and courting males (*L. hesperus*: Wilks’ lambda = 0.05, *F*_8,84_ = 35.5, *p* < 0.001; *T. agrestis*: Wilks’ lambda = 0.19, *F*_8,84_ = 13.29, *p* < 0.001). There was only slight overlap in the vibration parameters from each source (Figure 6). In *L. hesperus*, only 12.5% of vibrations were misclassified, and no male vibrations were misclassified as prey. In *T. agrestis*, only 16.7% of vibrations were misclassified; two male vibrations were misclassified as fly vibrations, and three fly vibrations were misclassified as male vibrations. On *L. hesperus* webs, the source of vibrations had a significant effect on the dominant frequency (*F*_2,45_ = 5.8, *p* = 0.0057), RMS amplitude (*F*_2, 45_ = 18.83, *p* = 0.0001), and the duration (*F*_2, 45_ = 128.27, *p* < 0.0001) of vibrations. Post-hoc analyses revealed that vibrations of *L. hesperus* males have a mean dominant frequency twice as high as that of crickets and flies, while the mean duration of male vibrations is four to five times longer than those of flies or crickets. The amplitude of male *L. hesperus* vibrations was not significantly different from that of cricket vibrations, but was lower than that of fly vibrations (Figure 6).

On *T. agrestis* webs, the source of vibrations had a significant effect on the amplitude (*F*_2, 45_ = 7.57, *p* = 0.0015) and the duration (*F*_2, 45_ = 52.52, *p* < 0.0001) of vibrations but not on their dominant frequency (*F*_2, 45_ = 0.35, *p* = 0.71). Post-hoc analyses showed that vibrations of *T. agrestis* males last on average two to three times longer than those of flies or crickets. The amplitude of male vibrations was not significantly different from that of either fly or cricket vibrations (Figure 6).

We did not perform ANOVAs on bandwidth because we found this variable to be highly correlated with dominant frequency for crickets, flies and males in both species (*L. hesperus*: Pearson correlation coefficient *r* = 0.76, *p* < 0.0001, *n* = 48; *T. agrestis*: *r* = 0.65, *p* < 0.0001, *n* = 48).

### Vibration parameters triggering a predatory response in *L. hesperus* females

**Figure 7:**
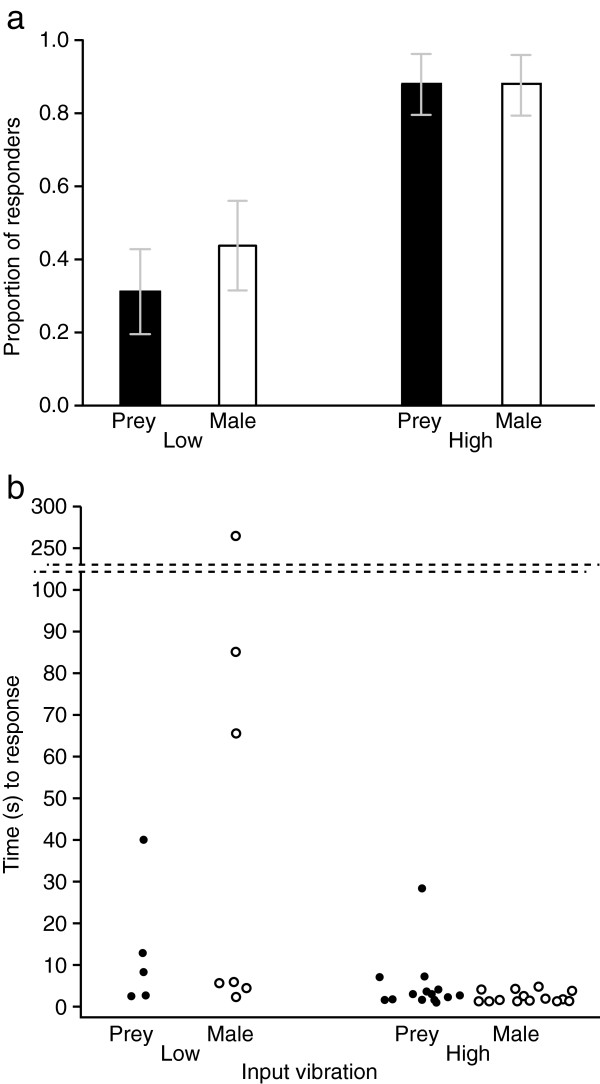
**Response of female *Latrodectus hesperus *to playback of high- and low-amplitude prey and male vibrations. (a)** Proportion of *Latrodectus hesperus* females responding aggressively to vibrations produced by house fly prey or conspecific males played back at high or low amplitude at a distance of 15 cm (n = 16 for each treatment). Whiskers = standard error; **(b)** Time [s] elapsed before females initiated a predatory response (number of responding females: n = 5 for prey/low, n = 7 for male/low, n = 14 for both prey/high and male/high).

We tested the response of *L. hesperus* females to playback of prey and male vibrations presented at low and high amplitude. The amplitude, but not the waveform, of vibrations had a significant effect on the behavioural response of *L. hesperus* females (amplitude: χ^2^_1,64_ = 14.41, *p* = 0.0001; waveform: χ^2^_1,64_ = 0.17, *p* = 0.679). Far fewer females responded to the ‘low’ amplitude stimulus (‘prey’ waveform: 31.25%; ‘male’ waveform: 43.75%) than to the ‘high’ amplitude stimulus (‘prey’ and ‘male’ waveform: 87.5%). The interaction term between amplitude and waveform was not significant (χ^2^_1,64_ = 0.17, *p* = 0.679). The proportion of females that exhibited a predatory response to playbacks of low- or high-amplitude prey cues and low- or high-amplitude male signals is shown in Figure 7a, and results of the logistic regression analysis for the full factorial model (χ^2^_3,64_ = 18.762, *p* = 0.0003) are presented in Table 2.

**Table 2:** **Results of logistic regression analysis of predatory responses of female *Latrodectus hesperus *** **to playbacks of low- or high-amplitude vibrations of house fly prey and conspecific males**

**Model**	**- LogLikelihood**		**DF**	**χ**^**2**^	***p***
Difference	9.381		3	18.762	0.0003
Full	32.959				
Reduced	42.340				
*R*^2^ (U)	0.222				
**Predictor**	**β**	**SE β**	**χ**^**2**^	***p***	**Odds ratio**
Constant	- 0.713	0.325	4.82	0.028	N/A
Amplitude	1.233	0.325	14.41	0.0001	1.308
Waveform	- 0.134	0.325	0.17	0.679	0.085
Amplitude*Waveform	- 0.134	0.325	0.17	0.679	N/A

(The levels of the factors amplitude and waveform were coded as follows: low = 0, high = 1; male = 0, prey = 1).

Among the females exposed to playback recordings of ‘low’ amplitude ‘prey’ or ‘male’ vibrations, seven females remained immobile. In response to the ‘low prey’ stimulus, two females twitched their abdomen and four females moved their legs or oriented toward the source of the vibration. In response to the ‘low male’ stimulus, one female displayed an abdomen twitch and two females adjusted the position of their legs or oriented toward the source of the vibration. The only two females who did not exhibit a predatory response to the playback of a ‘high’ amplitude ‘prey’ or ‘male’ vibration remained immobile.

For females that responded to the playback stimulus, the latency of their response to the four test stimuli (see Materials and Methods) is reported in Figure 7b. Results of the survival analysis show that there was no significant difference between the response times of females exposed to playback of low-amplitude prey or male vibrations (Wilcoxon test; χ^2^_1,32_ = 0.2921, *p* = 0.589), and no significant difference between the response times of females to playback of high-amplitude prey or male vibrations (Wilcoxon test; χ^2^_1,32_ = 2.3105, *p* = 0.129). Most females responded very quickly to both high-amplitude stimuli and started moving toward the source of the vibration in less than 10 s. Far fewer females responded to the low-amplitude treatments, and they did so more slowly.

## Discussion

We have (1) characterized vibratory cues of house fly and house cricket prey and vibratory signals of *L. hesperus* and *T. agrestis* males; (2) determined that vibratory courtship signals of males differ from prey vibratory cues*;* and (3) ascertained the vibration parameter(s) triggering a predatory response in females.

On both *L. hesperus* and *T. agrestis* webs, cricket and fly vibrations were similar: short, sporadic, and on average with high amplitude modulation. Most vibrations of *L. hesperus* and *T. agrestis* males were continuous, lengthy, and lacking a complex temporal structure. Vibrations of *L. hesperus* males differed from prey in terms of duration and dominant frequency. Male vibrations were of lower amplitude than fly, but not cricket, vibrations. Vibrations of *T. agrestis* males differed from prey in terms of duration only. During the playback experiment, significantly fewer *L. hesperus* females responded aggressively to low-amplitude vibrations, irrespective of whether these stimuli were recorded vibrations of prey or male spiders, suggesting that the likelihood of a predatory response depends on the amplitude but not the waveform of incoming vibrations. Below we discuss the implications of these findings for male signal function and signalling constraints.

The absence of complex temporal patterns in most courtship vibrations of *L. hesperus* and *T. agrestis* males is in stark contrast to observations in other spiders. For example, female *Cupiennius getazi* use the duration and structure of male-produced syllables to identify conspecific males [29]. Males of the wolf spider *Lycosa tarentula fasciiventris* produce courtship vibrations that comprise series of repeating syllables followed by pauses at regular intervals [30]. The temporal structure of courtship vibrations produced by male *Schizocosa ocreata* is linked to female mate choice [31]. Finally, the vibratory courtship displays of 11 species of jumping spiders (Salticidae) within the *Habronattus coecatus* clade are complex and comprise up to 20 distinct elements organized in motifs [32]. All of the above examples refer to wandering spiders whose courtship takes place on plant stalks, leaf litter, or the ground. Similarly, courting males of the orb-weaver *Argiope keyserlingi* produce vibrations with repeated, pulse-like characteristics [18]. This distinct temporal patterning may be well transmitted because *A. keyserlingi* males court on a single silk thread. The resulting vibrations are quite different from the ones reported in our study, but the abdominal tremulation of *L. hesperus* males and the shuddering of *A. keyserlingi* males are very similar types of behaviour. The absence of temporally complex signalling in *L. hesperus*, and its scant presence in *T. agrestis*, is curious. Based on their rate and amplitude modulation, tremulations can produce signals with a lot of information [33] but abdominal tremulations of male *L. hesperus* generated uniform waveforms that can be described solely on the basis of their amplitude and frequency. Future work is needed to reveal whether sheet and tangle webs impose constraints on the temporal complexity of signals.

The transmission properties of a medium impose constraints on the characteristics of signals [1,34]. Contrary to orb webs, *L. hesperus* tangle webs and *T. agrestis* sheet webs are not uniform structures. The density of threads and their orientation, degree of tension, number of connections and distance to anchor points all vary greatly from one area of the web to another, and likely affect the transmission characteristics of the webs. When we explored the propagation properties of transversal vibrations on *L. hesperus* and *T. agrestis* webs (using frequency sweeps from 0 to 500 Hz), we found great variability both within and between webs in both types of webs (S. Vibert, unpublished data). Within a single web, transmission profiles obtained at different locations were sometimes very dissimilar. Similarly, playback of recorded prey vibrations of known dominant frequency revealed that frequency was not well transmitted across *L. hesperus* webs (S. Vibert, unpublished data).

There are several plausible explanations for the difference between male and prey vibrations. Vibrations on webs during courtship interactions might communicate species identity and help females distinguish between con- and heterospecific males or between conspecific males and potential prey. Alternatively, vibrations of a male might communicate his identity, quality, or current location.

Our results suggest that the low-amplitude vibrations produced by *L. hesperus* males reduce the probability of being attacked by females during courtship. Female attack rate was twice as low when prey or male vibrations were played back at the low amplitude of male abdominal tremulations than at the high amplitude of prey vibrations. We also observed females twitching their abdomen dorso-ventrally in response to three low-amplitude playbacks. In a previous study [20], 75% of *L. hesperus* females displayed “twitching” during advanced stages of courtship, whereas no female ever displayed twitching in response to live prey (S. Vibert, pers. obs.). Our results do suggest that *L. hesperus* males must “whisper” during courtship, but the potential information content and sexual function of these whispers are yet be studied. It would be particularly interesting to investigate whether *T. agrestis* females respond differently to vibrations of varying duration, the one parameter in which vibrations of males differed from those of prey. While prey vibrations were intermittent, vibrations of *L. hesperus* and *T. agrestis* males were continuous, which may be another determinant factor for the females’ predatory responses.

The function of *L. hesperus* male vibratory signals is not likely to advertise male quality. Whenever males deploy acoustic signals that broadcast their quality, females prefer loud (high-amplitude) signals, as has been demonstrated in gray tree frogs [35], túngara frogs [36], katydids [37], wax moths [38], and passerine birds [39,40]. Whether the amplitude of vibratory signals produced by courting spider males is indicative of their quality as prospective mates, or whether it serves another function, has hardly been studied. Large males of the funnel-web spider *Agelenopsis aperta* are more likely to achieve mating success [6], possibly because they produce louder signals, as has been shown for airborne signals in the toad *Bufo americanus*[41]. Similarly, male *Schizocosa ocreata* wolf spiders producing higher-amplitude signals were more successful at securing a mate [31]. In the wandering spider *Cupiennius*, however, the amplitude of signals seems of no relevance to females [42-44]. The quiet songs of birds exemplify a signalling display characterized by low amplitude; quiet songs prevent eavesdropping from competitors or rivals in contexts of territorial disputes or mating interactions [45]. The courtship display of *L. hesperus* might well represent a novel context in which males must signal at low amplitude to avoid triggering a female predatory response.

A reduction of female aggressiveness is often cited as one of the possible functions of male courtship in spiders but few studies have tested this experimentally. Many adaptations may function to avoid or reduce female aggressiveness. Behavioural adaptations include approaching a female while she is feeding [46], mate binding [47], or inducing a quiescent state [6]. We suggest that courtship signals of *L. hesperus* and *T. agrestis* males that differ markedly from prey vibrations might represent another adaptation in males facing large and aggressive females. Conversely, in species where females are not aggressive towards males, it may be adaptive for courting males to take advantage of the females’ sensory systems being tuned for prey cues by producing prey-like vibrations, as has been demonstrated in the water mite *Neumania papillator*[48].

## Conclusions

Silk production is one of the most fascinating innovations of spiders, aiding in many aspects of their natural history. The use of webs in prey capture is well documented. Less studied is how males communicate through the females’ webs during courtship displays. We present an exploratory study of vibratory courtship signals on tangle and sheet webs. In both *L. hesperus* and *T. agrestis,* we found that some parameters of male courtship signals contrast with those of prey cues. In *L. hesperus,* one of these parameters seems to facilitate male courtship in that *L. hesperus* females are less likely to attack in response to the characteristic low-amplitude vibrations of *L. hesperus* males than in response to the high-amplitude vibrations of prey. Many other aspects are yet to be investigated in future studies. They include signal transmission properties of the highly complex and variable tangle and sheet webs, their potential constraints on male signal design, the information content of male signals, and their role in eliciting a sexual response from females.

## Competing interests

The authors declare that they have no competing interests.

## Authors’ contributions

SV conceived of the study and drafted the manuscript. SV and CS designed the study, collected the study animals, performed the research and statistical analyses. CS and GG critically revised the manuscript. All authors read and approved the final manuscript.

## Supplementary Material

Additional file 1 Abdominal tremulation of black widow male. Click here for file

Additional file 2 Walking, drumming and tapping of hobo spider 725 male. Click here for file

Additional file 3 Jerk of hobo spider male. Click here for file

Additional file 4 **Background noise recordings on *Latrodectus hesperus *** **and *Tegenaria agrestis *** **webs.** Click here for file

Additional file 5 Number of vibration recordings and number of events within six randomly selected recordings for each replicate. Click here for file

Additional file 6 Waveform quality and amplitude consistency of playback-induced vibrations. Click here for file

Additional file 7 Playback design, input and playback-induced vibrations, and box-plots of the root mean square amplitude and amplitude modulation factor of the playback-induced vibrations. Click here for file

Additional file 8 Aggressive response of a black widow female to a male high-amplitude playback vibration. Click here for file
