# Supplementary material for: A meal or a male: the ‘whispers’ of black widow males do not trigger a predatory response in females
Source: Front Zool. 2014 Jan 17;11:4. doi: 10.1186/1742-9994-11-4 (PMC3909478; doi:10.1186/1742-9994-11-4)
Supplement: Additional file 4 — Background noise recordings on Latrodectus hesperus and Tegenaria agrestis webs. [file 1742-9994-11-4-S4.pdf]

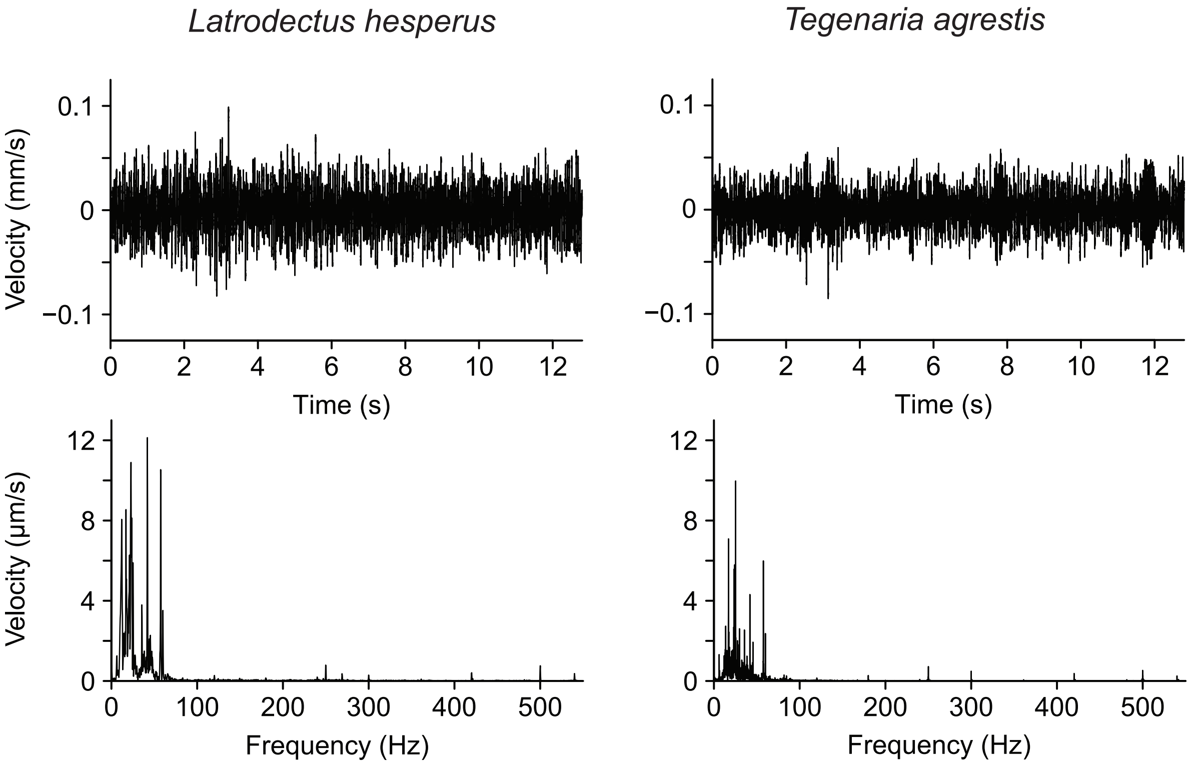

Additional file 4. Background noise recordings on *Latrodectus hesperus* and *Tegenaria agrestis* webs.
Representative recordings of background noise associated with empty webs. Oscillograms depict velocity [mm/s] over time [s] (upper panel) and velocity [µm/s] over frequency [Hz] (lower panel).
