# Supplementary material for: A meal or a male: the ‘whispers’ of black widow males do not trigger a predatory response in females
Source: Front Zool. 2014 Jan 17;11:4. doi: 10.1186/1742-9994-11-4 (PMC3909478; doi:10.1186/1742-9994-11-4)
Supplement: Additional file 5 — Number of vibration recordings and number of events within six randomly selected recordings for each replicate. [file 1742-9994-11-4-S5.pdf]

Additional file 1. Number of recordings and number of events within 6 randomly selected recordings for each replicate.

Mean, standard deviation (SD), min. and max. number (#) of 12.8-s laser Doppler vibrometer recordings of (*i*) vibrations produced by courting males, house flies, and crickets on empty webs of female *Latrodectus hesperus* (*Lh*) and *Tegenaria agrestis* (*Ta*) and (*ii*) 'events' measured within six randomly selected recordings for each male, fly, and cricket replicate. 'Events' are segments of a recording with a vibration amplitude > 2× background amplitude.

		**Recordings per replicate**			
		Mean	SD	Min. #	Max. #
Lh	Male	32.1	11.0	16	52
	Fly	17.6	8.1	6	38
	Cricket	17.9	6.4	6	26
Ta	Male	24.7	11.7	6	46
	Fly	12.9	3.9	6	20
	Cricket	15.9	1.7	14	20
		**Events per replicate within 6 recordings**			
		Mean	SD	Min. #	Max. #
Lh	Male	7.9	1.7	6	11
	Fly	12.0	4.0	7	22
	Cricket	10.9	3.6	7	17
Ta	Male	10.4	3.5	7	17
	Fly	12.6	4.6	6	25
	Cricket	12.7	4.0	7	21

Number of replicates for males, flies, and crickets = 16 for both *Lh* and *Ta*.
