# Supplementary material for: A meal or a male: the ‘whispers’ of black widow males do not trigger a predatory response in females
Source: Front Zool. 2014 Jan 17;11:4. doi: 10.1186/1742-9994-11-4 (PMC3909478; doi:10.1186/1742-9994-11-4)
Supplement: Additional file 6 — Waveform quality and amplitude consistency of playback-induced vibrations. [file 1742-9994-11-4-S6.pdf]

**Additional file 2. Waveform quality and amplitude consistency of playback-induced vibrations**

To assess waveform fidelity after transmission through the web as well as amplitude consistency of playback-induced vibrations, we played back the ‘prey’ and ‘male’ vibrations at ‘low’ and ‘high’ levels on nine 21-day-old empty female webs and recorded the resulting playback-induced vibrations at a distance of 15 cm from the input location of the playback vibrations (Additional file 7a). We then measured the peak-to-baseline amplitude of the playback-induced vibrations. At the ‘low’ level, prey and male playback vibrations induced a mean peak-to-baseline vibration amplitude of 2.14 mm/s (1.35 S.D.) and 1.83 mm/s (1.39 S.D.), respectively. At the ‘high’ level, male and prey playback vibrations induced a mean vibration amplitude of 8.32 mm/s (2.80 S.D.) and 10.61 mm/s (4.53 S.D.), respectively. Equipment limitations and increasing distortion of vibration waveforms with increasing amplification prevented us from raising the high level to the desired amplitude of 21 mm/s (mean amplitude of fly vibrations). We conducted a two-way ANOVA with maximum (peak-to-peak) amplitude as the response variable and waveform (‘prey’ or ‘male’) and amplitude level (‘high’ or ‘low’) as fixed effect factors, using web number as a blocking factor. Amplitude differed significantly between the high- and low-amplitude level treatments ($F_{3,24} = 94.59$, $p < 0.0001$) but not between the prey and male treatments ($F_{3,24} = 1.96$, $p = 0.17$). There was no significant interaction between waveform and amplitude level ($F_{3,24} = 3.25$, $p = 0.08$) (see Additional file 7-d). These measurements indicate that playback-induced vibration amplitudes were consistently transmitted at a distance of 15 cm from the input location.

To determine whether the waveform of vibrations is conserved after transmission through 15 cm of web we measured the amplitude modulation factor (AMF) of the resulting waveforms. Before proceeding with statistical analysis, we log-transformed the data to meet the assumption of equal variance. We conducted a two-way ANOVA with amplitude modulation factor (AMF) as the response variable and waveform (‘prey’ or ‘male’) and amplitude level (‘high’ or ‘low’) as fixed effect factors; web number was used as a blocking factor. The back-transformed marginal mean AMF values for the ‘male’ and ‘prey’ waveforms were 3.46 (*SE* 1.13) and 16.76 (*SE* 1.13), respectively. AMF differed significantly between the ‘male’ and ‘prey’ waveforms ($F_{3,24} = 95.79$, $p < 0.0001$). There was also a significant difference in AMF between the ‘low’ and ‘high’ amplitude levels ($F_{3,24} = 6.38$, $p = 0.019$). There was no significant interaction between waveform and amplitude level ($F_{3,24} = 0.10$, $p = 0.76$) (see Additional file 7-e). Although the waveform of playback-induced vibrations was not transmitted with perfect fidelity through 15 cm of web (as reflected by the wide range of AMF values for the ‘prey’ waveform), we observed very little overlap in terms of AMF between our ‘male’ and ‘prey’ treatment. Most (88%) ‘male’ playback-induced AMFs ranged between 2 and 6, and all ‘prey’ playback-induced AMFs were > 7.5. This indicates that the unique characteristics of the ‘prey’ and ‘male’ vibrations were consistently conserved in the playback-induced vibrations.
