# Supplementary material for: A meal or a male: the ‘whispers’ of black widow males do not trigger a predatory response in females
Source: Front Zool. 2014 Jan 17;11:4. doi: 10.1186/1742-9994-11-4 (PMC3909478; doi:10.1186/1742-9994-11-4)
Supplement: Additional file 7 — Playback design, input and playback-induced vibrations, and box-plots of the root mean square amplitude and amplitude modulation factor of the playback-induced vibrations. [file 1742-9994-11-4-S7.pdf]

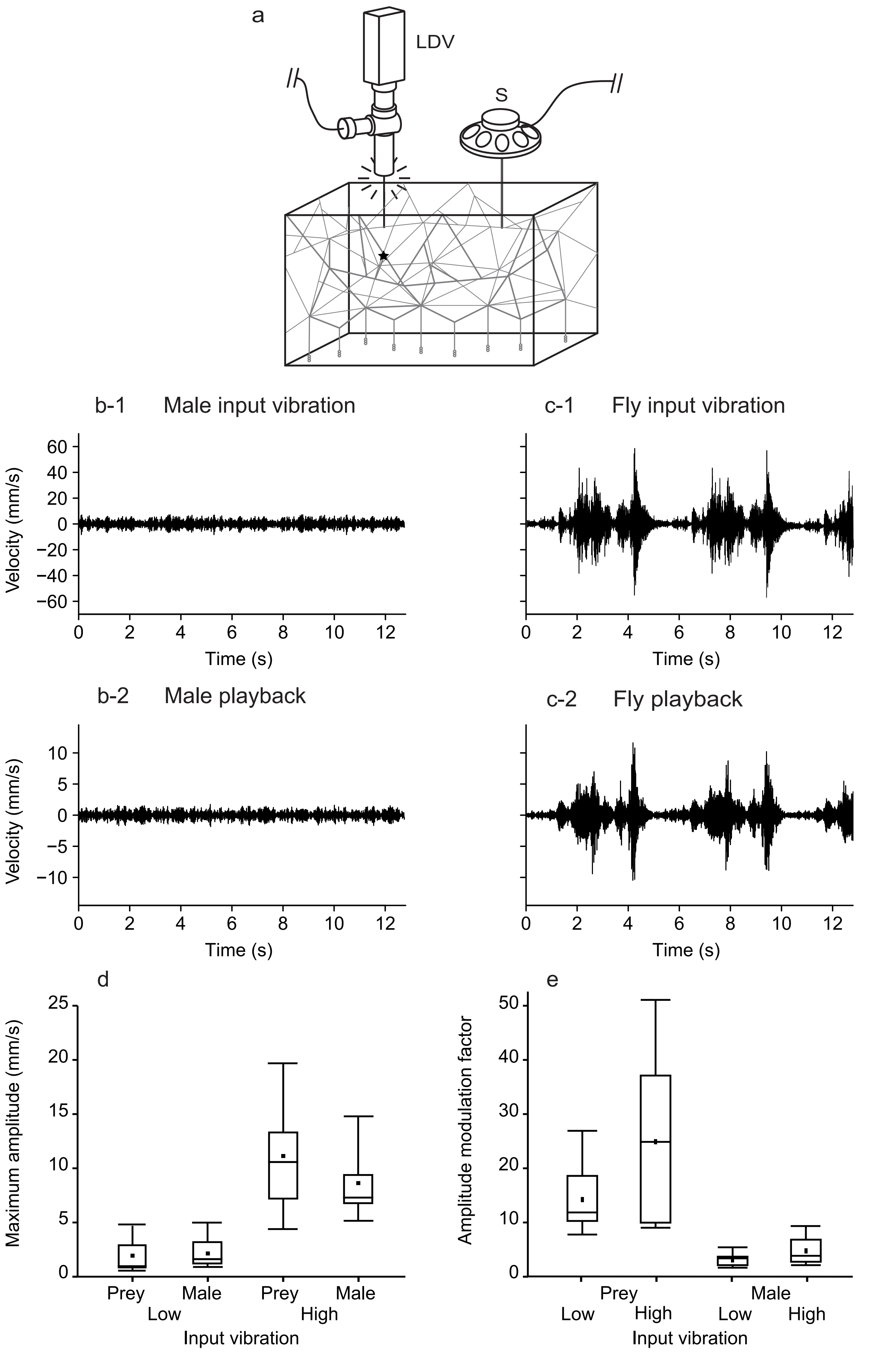

Additional file 7. Playback design, input and playback-induced vibrations, and box-plots of the root mean square amplitude and amplitude modulation factor of the playback-induced vibrations.

(a) Schematic drawing of the experimental design for vibration playbacks. A looped abdomen tremulation vibration of a male *Latrodectus hesperus* and a looped house fly vibration were played back on empty webs at low and high amplitude by a modified loudspeaker (S) (see main text for details), and transmission of playback-induced vibrations were recorded by a laser Doppler vibrometer (LDV) at a distance of 15cm. The star (★) indicates the recording location; (b-1 and b-2) Oscillograms of the looped male input vibration played back at low amplitude level, and a well-transmitted low-amplitude male playback-induced vibration; (c-1 and c-2) Oscillograms of the looped prey input vibration played back at high amplitude level, and a well-transmitted high-amplitude prey playback-induced vibration; note the difference in amplitude between input vibrations and playback-induced vibrations; (d) Box plots of the maximum amplitude of playback-induced prey and male vibrations at high- and low-amplitude level (n = 9 webs) recorded 15 cm away from the location of the input vibration. Median, mean and interquartile range (IQR); whiskers = upper and lower data point values within 1.5 IQR; (e) Box plots of the amplitude modulation factor of playback-induced prey and male vibrations at high-and low-amplitude level (n = 9 webs) recorded 15 cm away from the location of the input vibration. Median, mean and IQR; whiskers = upper and lower data point values within 1.5 IQR.
